# Secreted Frizzled-Related Protein 1 Promotes Odontoblastic Differentiation and Reparative Dentin Formation in Dental Pulp Cells

**DOI:** 10.3390/cells10092491

**Published:** 2021-09-21

**Authors:** Keita Ipposhi, Atsushi Tomokiyo, Taiga Ono, Kozue Yamashita, Muhammad Anas Alhasan, Daigaku Hasegawa, Sayuri Hamano, Shinichiro Yoshida, Hideki Sugii, Tomohiro Itoyama, Marina Ogawa, Hidefumi Maeda

**Affiliations:** 1 Department of Endodontology and Operative Dentistry, Division of Oral Rehabilitation, Faculty of Dental Science, Kyushu University, Fukuoka 812-8582, Japan; ipposhi@dent.kyushu-u.ac.jp (K.I.); tono@dent.kyushu-u.ac.jp (T.O.); kozue@dent.kyushu-u.ac.jp (K.Y.); anas@dent.kyushu-u.ac.jp (M.A.A.); shamano@dent.kyushu-u.ac.jp (S.H.); itoyama@dent.kyushu-u.ac.jp (T.I.); mogawa@dent.kyushu-u.ac.jp (M.O.); hide@dent.kyushu-u.ac.jp (H.M.); 2 Department of Endodontology, Kyushu University Hospital, Fukuoka 812-8582, Japan; daigaku8@dent.kyushu-u.ac.jp (D.H.); s.yosida@dent.kyushu-u.ac.jp (S.Y.); sugii@dent.kyushu-u.ac.jp (H.S.); 3 OBT Center, Faculty of Dental Science, Kyushu University, Fukuoka 812-8582, Japan

**Keywords:** SFRP1, dental pulp cells, dentinogenesis, direct pulp capping, reparative dentin

## Abstract

Direct pulp capping is an effective treatment for preserving dental pulp against carious or traumatic pulp exposure via the formation of protective reparative dentin by odontoblast-like cells. Reparative dentin formation can be stimulated by several signaling molecules; therefore, we investigated the effects of secreted frizzled-related protein (SFRP) 1 that was reported to be strongly expressed in odontoblasts of newborn molar tooth germs on odontoblastic differentiation and reparative dentin formation. In developing rat incisors, cells in the dental pulp, cervical loop, and inner enamel epithelium, as well as ameloblasts and preodontoblasts, weakly expressed Sfrp1; however, Sfrp1 was strongly expressed in mature odontoblasts. Human dental pulp cells (hDPCs) showed stronger expression of *SFRP1* compared with periodontal ligament cells and gingival cells. *SFRP1* knockdown in hDPCs abolished calcium chloride-induced mineralized nodule formation and odontoblast-related gene expression and decreased *BMP-2* gene expression. Conversely, SFRP1 stimulation enhanced nodule formation and expression of *BMP-2*. Direct pulp capping treatment with SFRP1 induced the formation of a considerable amount of reparative dentin that has a structure similar to primary dentin. Our results indicate that SFRP1 is crucial for dentinogenesis and is important in promoting reparative dentin formation in response to injury.

## 1. Introduction

The tooth is composed of four different types of dental tissue: enamel, dentin, cementum, and pulp. All of these tissues are calcified except for the pulp, which contains connective tissues, blood vessels, and nerves. Connective tissues in the pulp are loose and highly specialized, and show specific responses to trauma and microbial insults [1]. As in other tissues, blood vessels in the pulp provide nutrients and oxygen, regulate the immune response, and remove unnecessary substances [2]. The main function of nerves in the pulp is sensory; however, they also support pulp defense by promoting the extravasation of immune cells [3]. Therefore, the pulp is considered to play essential roles in tooth maintenance and homeostasis.

When the pulp is irreversibly infected or necrotic due to caries, traumatic injury, or other cause, dentists often perform endodontic treatment to remove all of the pulp in the pulp chamber and root canal. However, endodontic treatment sometimes leads to tooth discoloration causing esthetic problems. Moreover, pulp-extracted teeth are associated with vertical root fractures [4,5]. Vertical root fractures are an untoward complication that has a poor prognosis and often results in tooth extraction. Therefore, preserving pulp tissue is of great importance for tooth preservation and esthetics.

Direct pulp capping is a treatment to preserve pulp vitality in cases of carious or traumatic pulp exposure. In this method, a healing agent is placed directly over the exposed pulp to promote the formation of protective reparative dentin and to maintain vital pulp. Various materials have been used in direct pulp capping and two types of material, calcium hydroxide (CH) and mineral trioxide aggregate (MTA), are now most commonly used because they show high biocompatibility, good sealing capacity, antibacterial effects, and promote mineralized tissue formation [6]. However, CH can gradually disintegrate and has sub-optimal mechanical properties, and MTA has a long setting time and is difficult to handle [7,8]. In addition, newly formed hard tissues derived from these materials do not resemble reparative dentin because they include porosities and tunnel defects [9]. Therefore, the development of a new pulp capping material is required to induce the formation of hard tissues that highly resemble mature reparative dentin.

Primary dentin constitutes the majority of the dentin mass and is produced by odontoblasts. These cells have the potential to generate an organic matrix that is capable of mineralization during initial dentinogenesis. Reparative dentin is produced by odontoblast-like cells that are derived from the mesenchymal stem or progenitor cells in pulp in response to injuries [10]. Dental pulp-derived stem cells are attracting much attention today due to their strong differentiation potential and availability. To enhance the effectiveness of stem cells in regenerative treatment, some studies are actively conducted on the interactions between secretome and scaffolding materials [11,12]. Many reports also have demonstrated the involvement of signaling molecules in the induction of reparative dentin formation [13,14]. The secreted frizzled-related protein (SFRP) family acts as a canonical Wnt signaling inhibitor either by sequestering canonical Wnt ligands or by forming nonfunctional complexes with Frizzled receptors. This family is composed of five secreted glycoproteins, SFRP1, SFRP2, SFRP3 (Frzb), SFRP4, and SFRP5, which have been identified in humans, mice, and chickens [15]. SFRP1, a 35 kDa prototypical member of the SFRP family, has a role in regulating cell growth and differentiation of specific cell types and contributes to development and homeostasis in various tissues [15]. SFRP1 is expressed in osteogenic cells, such as osteoblasts and chondrocytes, and its expression level increases as their differentiation advances [16]. SFRP1 is also strongly expressed in odontoblasts of newborn molar tooth germs [17]; however, the effects of SFRP1 on odontoblastic differentiation and on the formation of reparative dentin have not been elucidated.

The aim of this study was to determine whether SFRP1 can promote odontoblastic differentiation of dental pulp cells and reparative dentin formation after direct pulp capping treatment.

## 2. Materials and Methods

### 2.1. Immunohistochemical Staining

Our animal study was carried out in compliance with the ARRIVE guidelines, and all procedures were approved by the Animal Ethics Committee and conformed to the regulations of Kyushu University (A30-342-0, 15 February 2019). Eight-week-old male Wistar rats (Kyudo, Saga, Japan) were perfused transcardially with 4% paraformaldehyde (PFA; Nacalai Tesque, Kyoto, Japan) in phosphate-buffered saline (PBS) under anesthesia. Their maxillae and mandibles were excised and immersed in 4% PFA for an additional 24 h. These samples were decalcified using 10% ethylenediaminetetraacetic acid (EDTA; Nacalai Tesque) at 4 °C for 1 month before embedding in paraffin. The tissues were sectioned at a thickness of 5  μm. After deparaffinization, the sections were blocked with 2% bovine serum albumin (BSA; Nacalai Tesque) in PBS for 1  h at room temperature and then incubated with a rabbit polyclonal anti-rat Sfrp1 antibody (1:50; Proteintech, Chicago, IL, USA), or normal rabbit immunoglobulin G (IgG) (Cell Signaling Technology, Beverly, MA, USA) overnight at 4 °C. Following incubation with biotinylated anti-rabbit IgG (Nichirei Biosciences, Tokyo, Japan) for 30  min at room temperature, sections were reacted with an avidin-peroxidase conjugate (Nichirei Biosciences) for 30 min at room temperature. Simple Stain DAB solution (Nichirei Biosciences) visualized positive staining. Nuclei were stained using Mayer’s hematoxylin solution (FUJIFILM wako pure chemical corporation, Osaka, Japan). Sections were observed using an inverted microscope (BX41; Olympus, Tokyo, Japan).

### 2.2. Cell Culture

All in vitro procedures were carried out following the rules of the Declaration of Helsinki and in accordance with the requirements of the Research Ethics Committee of Kyushu University (2–115, 10 August 2020) and informed consent was obtained from all tissue donors. In the case of 5I, informed consent was obtained from the patient’s mother. Three hDPC, hPDLC, and hGF populations (5N (24-year-old male), 5I (16-year-old female), and 3R (24-year-old male)) were isolated from the healthy premolars or a third molar of patients who visited the Dental Hospital of Kyushu University for extractions, as described previously [18,19]. Briefly, PDL tissues were stripped from the root surface of the extracted teeth. Then, these teeth were cleaved and pulp tissues were isolated from them. Gum tissues were also obtained by cutting from the periphery of the extracted tooth. These tissues were incubated at 37 °C for 20 min in the presence of collagenase and trypsin. The dispersed cells were retrieved by centrifuging at 1000 rpm for 5 min. Cells from passages four through six were used as hDPC, hPDLC, and hGF in this study.

### 2.3. Semi-Quantitative Reverse Transcription Polymerase Chain Reaction

hDPCs, hPDLCs, and hGFs were cultured in 60-mm dishes (1 × 10^5^ cells/dish) for 24 h in control medium (CM) composed of alpha minimal essential medium (α-MEM; Gibco-BRL, Grand Island, NY, USA) containing 10% fetal bovine serum (FBS; Sigma-Aldrich, St. Louis, MO, USA). Following total RNA extraction and first-strand cDNA synthesis, semi-quantitative reverse transcription polymerase chain reaction (RT-PCR) was performed using Platinum Taq DNA polymerase (Invitrogen, Carlsbad, CA, USA) and a PCR Thermal Cycler Dice (Takara Bio, Shiga, Japan). PCR conditions were 94 °C for 2 min and then 94 °C for 30 s, appropriate annealing temperature for 30 s, 72 °C for 30 s for the appropriate number of cycles, and finally 72 °C for 7 min, in accordance with our recent study [16]. Primer sequences, annealing temperatures, cycle number, and product sizes for *SFRP1* and *Glyceraldehyde 3-phosphate dehydrogenase* (*GAPDH*) are listed in Table 1. *GAPDH* primers were used as internal standards. The RT-PCR products were separated by electrophoresis on 2% agarose gels (Seakem ME; BioWhittaker Molecular Applications, Rockland, ME, USA) containing ethidium bromide.

### 2.4. Odontoblastic Differentiation of hDPCs

We previously demonstrated that calcium chloride (CaCl_2_) can promote odontoblastic differentiation of hDPCs [20]. Therefore, we exposed hDPCs to CM, CM containing 2 mM CaCl_2_ (FUJIFILM wako pure chemical corporation) as odontoblastic differentiation medium (DM), or DM containing 100 ng recombinant human SFRP1 (rhSFRP1) (PeproTech, Rocky Hill, NJ, USA) (DM+SFRP1). hDPCs were seeded in 24-well plates with 3 × 10^4^ cells/well and cultured in CM, DM or DM+SFRP1 with media changes every 3 days. After 5 days of induction, cells were fixed with 10% formalin (FUJIFILM wako pure chemical corporation) for 1 h at room temperature for Alizarin Red S staining. Total RNA was also extracted from cells incubated for 3 days for quantitative real-time RT-PCR analysis.

### 2.5. Alizarin Red S Staining

The generation of calcified deposits was visualized by Alizarin Red S staining. Briefly, fixed cells were washed five times with deionized water and then incubated with 2% Alizarin Red S (pH 4.1–4.3; Sigma-Aldrich) for 1 h at room temperature. Following five washes with deionized water, image capture was performed using a Keyence BZ-9000 microscope (Keyence, Osaka, Japan). Four fields were randomly chosen for quantification of Alizarin Red S-positive area. Measurements were performed using BZ-X Analyzer Software (Keyence).

### 2.6. Small Interfering RNA Transfection

hDPCs were transfected with human SFRP1 small interfering RNA (siRNA) (MISSION siRNA, SASI_Hs01_00078720; Sigma-Aldrich) or human control siRNA (MISSION siRNA Universal Negative Control #1, SIC-001-10; Sigma-Aldrich) using Lipofectamine RNAiMAX (Invitrogen) according to the manufacturer’s instructions. In detail, hDPCs (1 × 10^4^ cells/well) were cultured in Opti-MEM I (Invitrogen) containing 10% FBS for 24 h. A siRNA-lipid complex, comprising 10 pmol siRNA and 3 µL Lipofectamine RNAiMAX in 50 µL Opti-MEM, was prepared. After incubation for 15 min at room temperature, the complex was added to the cells, and the cells were incubated for 24  h. siRNA-transduced hDPCs were cultured in CM or DM with media changes every 3 days. After 5 days of induction, cells were fixed with 10% formalin for 1 h at room temperature for Alizarin Red S staining. Total RNA was also extracted from cells incubated for 24 h or 2 days for quantitative real-time RT-PCR analysis.

### 2.7. Quantitative Real-Time Reverse Transcription Polymerase Chain Reaction

Total cellular RNA was isolated using TRIzol Reagent (Invitrogen) and reverse-transcribed using an ExScript RT reagent Kit (Takara Bio), according to the manufacturer’s instructions. First strand cDNA was synthesized from 1 μg total RNA. Total RNA was reverse-transcribed with random 6-mers and ExScript RTase for 15 min at 42 °C. The reaction was stopped by incubation for 2 min at 99 °C, followed by 5 min at 5 °C. Quantitative real-time RT-PCR was performed with KAPA Express Extract (Kapa Biosystems, Woburn, MA, USA) using a Thermal Cycler Dice Real Time System (Takara Bio) under the following conditions: 95 °C for 30 s and then 50 cycles of 95 °C for 5 s and 60 °C for 30 s, followed by a dissociation program at 95 °C for 15 s, 60 °C for 30 s, and 95 °C for 15 s. Specific primer sequences, annealing temperatures, and product sizes for each gene are listed in Table 2. β*-actin* was used as an internal control. Expression levels of target genes were calculated using ΔΔCt values.

### 2.8. Direct Pulp Capping Model

A direct pulp capping model was prepared as described previously [13]. Briefly, eight-week-old male Wistar rats (Kyudo) were anesthetized by intraperitoneal anesthesia composed of 2 mg/kg midazolam (Sandoz, Tokyo, Japan), 0.15 mg/kg medetomidine hydrochloride (Kyoritsuseiyaku, Tokyo, Japan), and 2.5 mg/kg butorphanol tartrate (Meiji Seika Pharma, Tokyo, Japan). A half-moon like cavity (1 mm in diameter and 1 mm in depth) was established on the mesial half of the occlusal surface of the upper left first molar using a No. 1/2 round steel bur (Dentsply Maillefer, Ballaigues, Switzerland). Then, pulp was exposed using a sterilized dental explorer. After the formation of pulp exposures, we measured their diameter and depth using a periodontal probe. The Nano β-TCP collagen scaffolds containing 100 or 200 ng rhSFRP1 were prepared to the same size as the artificially created cavities. They were gently applied to the pulp exposure site. Scaffolds containing PBS were used as a control. ProRoot MTA (Dentsply Sirona, Charlotte, NC, USA) was also used as direct pulp capping material. The cavity was then sealed with glass ionomer cement (Fuji IX; GC, Tokyo, Japan). After 2 or 4 weeks, the rats were transcardially perfused with 4% PFA (Nacalai Tesque) in PBS under anesthesia. Their excised maxillae were analyzed by a micro-CT scanner (SkyScan 1076; Bruker, Kontich, Belgium) at settings of 50 kV and 200 µA with a scanning resolution of 9 μm intervals in individual image. After scanning, three-dimensional reconstructed images of tissue (NRecon; Bruker, Kontich, Belgium) were used to analyze reparative dentin formation in each sample. After micro-CT scanning, the maxillae were decalcified using 10% EDTA at 4 °C for 1 month before embedding in paraffin. The tissues were sectioned at a thickness of 5 μm. After deparaffinization, the sections were stained with Mayer’s hematoxylin solution (FUJIFILM wako pure chemical corporation) for 100 s at room temperature. After washing with running tap water for 5 min, they were immersed in 0.1% Eosin solution (FUJIFILM wako pure chemical corporation) at room temperature for 3 min. Following washing with running with tap water for 1 min, the sections were observed using a BX41 microscope. The sections were also incubated with rabbit polyclonal anti-rat Nestin (Nes) antibody (1:100; Sigma-Aldrich), goat polyclonal anti-rat Dentin Sialoprotein (Dsp) antibody (1:200; Santa Cruz Biotechnology, Dallas, TX, USA), or normal rabbit immunoglobulin G (IgG) (Cell Signaling Technology) overnight at 4 °C. Following incubation with biotinylated secondary antibodies and an avidin-peroxidase conjugate, positive reactions were visualized using Simple Stain DAB solution. Nuclei were stained using Mayer’s hematoxylin solution. These specimens were observed using a BX41 microscope (*n* = 3 for each group). The amount of reparative dentin and the blockage rate of exposed pulp was quantified by Image J software (U. S. National Institutes of Health, Bethesda, MA, USA). This quantification was performed for reparative dentin in the pulp horn just below the exposed area in the hematoxylin and eosin (HE)-stained images.

### 2.9. Immunofluorescence Staining

hDPC-5I were fixed with 4% PFA (Nacalai Tesque) and 0.5% dimethyl sulfoxide (Wako) in PBS for 20 min at room temperature. After being blocked with 2% BSA in PBS for 1 h at room temperature, the cells were incubated with a rabbit polyclonal anti-β-catenin antibody (1:100; Cell Signaling Technology), as the primary antibody, or normal rabbit immunoglobulin G (IgG) (1:100; Cell Signaling Technology) overnight at 4 °C. The cells were then incubated with an Alexa 488-conjugated goat anti-rabbit IgG secondary antibody (1:200; Invitrogen) for 30 min at room temperature. The cells were then washed with PBS and counterstained with 4′,6-diamidino-2-phenylindole (DAPI; Nacalai Tesque). The cells were imaged and analyzed using a Biozero digital microscope (Keyence).

### 2.10. Western Blotting Analysis

hDPC-5I cultured in 10% FBS/αMEM, were treated with CM, DM, or DM+SFRP1 for 1 day. The cells were lysed in a buffer containing 50 mmol/L TRIS-HCl, pH 6.9 (Sigma-Aldrich), 2% sodium dodecylsulfate (SDS; Nacalai Tesque), 6% 2-mercaptoethanol (Sigma-Aldrich), and 10% glycerol. Aliquots containing 10 mg protein/lane were subjected to 4–20% SDS polyacrylamide gel electrophoresis and then transferred onto an Immuno-Blot PVDF membrane (Bio-Rad Laboratories, Hercules, CA, USA). After blocking with 5% skim milk (Yukijirushi, Tokyo, Japan) for 1 h at room temperature, the membrane was reacted with mouse monoclonal anti-β-actin (1:1000; Santa Cruz Biotechnology) or rabbit monoclonal anti-non-phospho (active) β-catenin (1:1000; Ser33/37/Thr41; D13A1; Cell Signaling Technology) overnight at 4 °C. These membranes were incubated with biotinylated anti-mouse IgG (Nichirei Biosciences) or anti-rabbit IgG (Nichirei Biosciences) for 1 h at room temperature, and then reacted with an avidin-peroxidase conjugate (Sigma-Aldrich) for 1 h at room temperature. After the membrane was washed thoroughly, the reactive bands were visualized using ECL Select Western Blotting Detection Reagent (GE Healthcare, Buckinghamshire, UK) and observed the reaction by Image Quant LAS 500 (GE Healthcare).

### 2.11. Statistical Analysis

All data are presented as the mean ± SD. Statistical analyses were performed by one-way ANOVA followed by Tukey’s test for multiple comparisons. Student’s unpaired t test was performed for the comparison of two mean values. *p* < 0.05 was considered statistically significant.

## 3. Results

### 3.1. Localization of Sfrp1 in Dental Tissues and Expression of SFRP1 mRNA in Dental Cells

**Figure 1:**
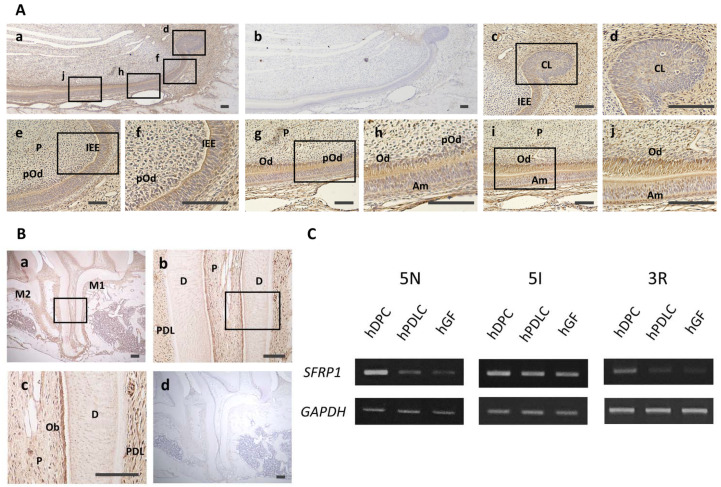
Localization of Sfrp1 in rat dental tissues and expression of SFRP1 mRNA in human dental cells. (**A**) Immunohistochemical staining of mandibular incisors of 8-week-old male Wistar rats with an anti-Sfrp1 antibody. Lower magnification images of anti-Sfrp1 (**a**) and rabbit control IgG (**b**) staining of mandibular incisor. (**c**,**e**,**g**,**i**) Higher magnification images in (**a**). (**d**,**f**,**h**,**j**) Higher magnification images of black boxes in (a,**c**,**e**,**g**,**i**). No positive staining was detected in the cervical loop (**c**,**d**). Faint positive staining was confirmed in the inner enamel epithelium (**e**,**f**), dental pulp (**e**,**f**), preodontoblast layer (**e**–**h**), and ameloblast layer (**g**,**h**). Intense positive staining was found in the odontoblast layer (**g**–**j**). (**B**) Immunohistochemical staining of mandibular first molar of 8-week-old male Wistar rats with an anti-Sfrp1 antibody (**a**–**c**) or rabbit control IgG (**d**). (**b**,**c**) Higher magnification images of black boxes in (**a**,**b**), respectively. Odontoblasts strongly expressed Sfrp1 compared with cells in the dental pulp and periodontal ligament. (**C**) Semi-quantitative RT-PCR analysis of SFRP1 gene expression in hDPCs, hPDLCs, and hGFs. In all three specimens, SFRP1 was strongly expressed in hDPCs compared with one in hPDLCs and hGFs. RT-PCR, reverse transcription polymerase chain reaction; hDPC, human dental pulp cells; hPDLC, human periodontal ligament cells; hGF, human gingival fibroblasts. Experiments were performed in duplicate. Representative data are shown. Bars = 100 μm. IgG, immunoglobulin G; CL, cervical loop; IEE, inner enamel epithelium; P, dental pulp; Am, ameloblast; pOd, preodontoblasts; Od, odontoblasts; M1, first molar; M2, second molar; D, dentin; PDL, periodontal ligament.

To assess Sfrp1 expression in odontoblasts at different stages of differentiation, we performed immunohistochemical staining of Sfrp1 in rat mandibular incisor tissues. Faint positive Sfrp1 staining was observed in the dental pulp, cervical loop, and ameloblast layer. Strong positive staining was observed in the odontoblast layer (Figure 1A). Cells in the dental pulp, cervical loop, and inner enamel epithelium expressed a low level of Sfrp1 (Figure 1A(c–f)). Ameloblasts and preodontoblasts also exhibited weak positive Sfrp1 staining (Figure 1A(g,h)); however, intense positive Sfrp1 staining was observed in odontoblasts (Figure 1A(g–j)). No staining was observed with control IgG (Figure 1A(b)). Immunohistochemical staining of mature dental tissues revealed positive staining for Sfrp1 in dental pulp, the odontoblast layer, and the periodontal ligament in rat maxillary first molar (Figure 1B(a–c)). Additionally, Sfrp1 levels were high in odontoblasts compared with cells in the dental pulp and periodontal ligament (Figure 1B(c)). No staining was observed with control IgG (Figure 1B(d)). Semi-quantitative RT-PCR analysis confirmed the expression of SFRP1 in hDPCs, hPDLCs, and hGFs isolated from three different patients (Figure 1C). Furthermore, hDPCs expressed higher levels of SFRP1, compared with hPDLCs and hGFs in all three specimens.

### 3.2. Expression of SFRP1 during Odontoblastic Differentiation of hDPCs

**Figure 2:**
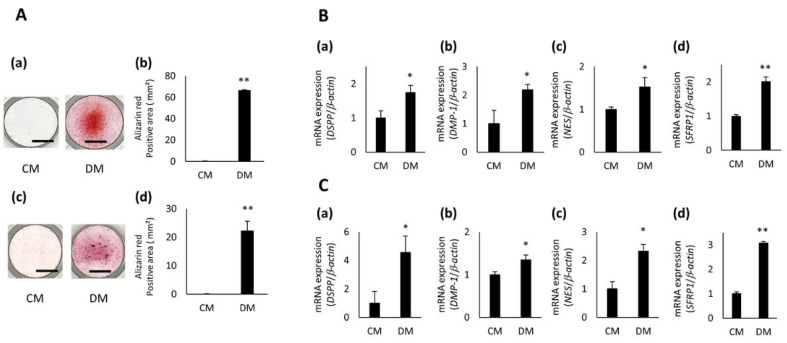
Gene expression of SFRP1 during odontoblastic differentiation of hDPCs. (**A**) Odontoblastic differentiation of hDPC-5N and -5I. Alizarin Red S staining images of hDPC-5N (**a**) and -5I (**c**) cultured in 10% FBS/α-MEM (control medium; CM) or CM with 2 mM CaCl_2_ (odontoblastic differentiation medium; DM) for 5 days. Experiments were performed in quadruplet. Representative data are shown. The Alizarin Red S-positive area of hDPC-5N (**b**) and -5I (**d**) cultured in CM or DM for 5 days. Gene expression of odontoblast related-markers, DSPP (**a**), DMP1 (**b**), NES (**c**), and SFRP1 (**d**) in hDPC-5N (**B**) and -5I (**C**) cultured in CM or DM for 3 days. Data are shown as the mean ± standard deviation (*n* = 3). * *p* < 0.05, ** *p* < 0.01. Scale bars = 5 mm. DSPP, dentin sialophosphoprotein; DMP1, dentin matrix acidic phosphoprotein 1; NES, Nestin.

Mineralized nodule formation was confirmed in all three hDPCs cultured in DM for 5 days; however, no nodule formation was observed in cells cultured in CM (Figure 2A(a,c) and Figure S1A(a)). hDPCs exposed to DM had a significantly larger Alizarin Red S stained area, compared with cells exposed to CM (Figure 2A(b,d) and Figure S1A(b)). In addition, the expression of odontoblast-related genes, dentin sialophosphoprotein (DSPP), dentin matrix acidic phosphoprotein 1 (DMP1), and Nestin (NES) was significantly up-regulated in hDPCs cultured in DM for 3 days compared with cells cultured in CM (Figure 2B(a–c),C(a–c) and Figure S1B(a–c)). Moreover, the expression level of SFRP1 was higher in hDPCs treated with DM than in cells treated with CM (Figure 2B(d),C(d) and Figure S1B(d)).

### 3.3. Effects of SFRP1 Down-Regulation on Odontoblastic Differentiation of hDPCs

**Figure 3:**
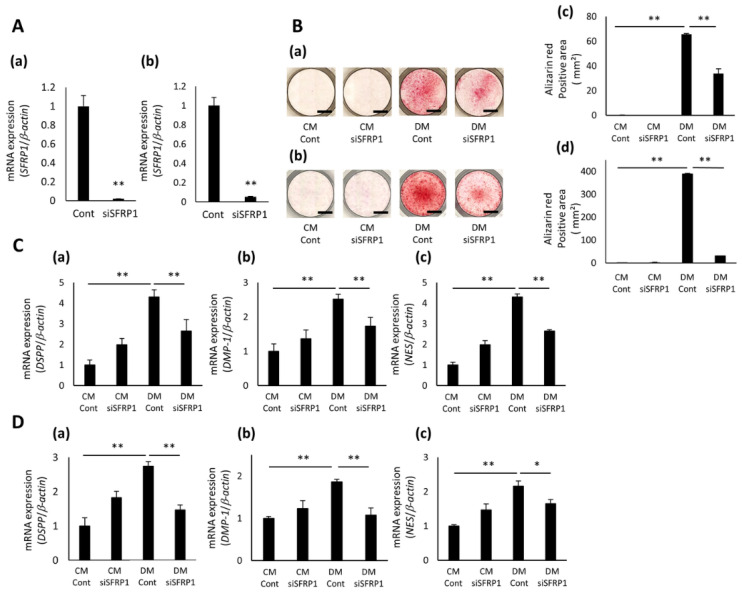
Effects of SFRP1 down-regulation on odontoblastic differentiation in hDPCs. (**A**) SFRP1 gene expression in hDPC-5N (**a**) and -5I (**b**) transduced with control siRNA (Cont) or SFRP1 siRNA (siSFRP1). (**B**) Odontoblastic differentiation of siRNA-transduced hDPC-5N and -5I. Images of Alizarin Red S staining of siRNA-transduced hDPC-5N (**a**) and -5I (**c**) cultured in CM or DM for 5 days. Experiments were performed in quadruplets. Representative data are shown. The Alizarin Red S-positive area of siRNA-transduced hDPC-5N (**b**) and -5I (**d**) cultured in CM or DM for 5 days. Gene expression of DSPP (**a**), DMP1 (**b**), and NES (**c**) in siSFRP1-transduced hDPC-5N (**C**) and -5I (**D**) cultured in CM or DM for 2 days. Data are shown as the mean ± standard deviation (*n* = 3). * *p* < 0.05, ** *p* < 0.01. Scale bars = 5 mm.

The expression of SFRP1 was significantly decreased in SFRP1 siRNA (siSFRP1)-transduced hDPCs, compared with that in control (Cont)-transduced cells (Figure 3A(a,b) and Figure S2A). Cont- or siSFRP1-transduced hDPCs cultured in CM for 5 days generated no mineralized nodules or Alizarin Red S-positive staining; however, these cells cultured in DM formed lots of nodules and stained positively with Alizarin Red S (Figure 3B(a–d) and Figure S2B(a,b)). In addition, siSFRP1-transduced hDPCs exposed to DM generated a lower number of mineralized nodules and a smaller Alizarin Red S-positive area than Cont-transduced cells exposed to DM (Figure 3B(a–d) and Figure S2B(a,b)). The expression of DSPP, DMP1, and NES was significantly up-regulated in Cont-transduced hDPCs cultured in DM for 2 days compared with these cells cultured in CM (Figure 3C(a–c),D(a–c) and Figure S2C(a–c)). However, the expression of these genes in siSFRP1-transduced hDPCs treated with DM was significantly decreased compared with that in Cont-transduced cells treated with DM (Figure 3C(a–c),D(a–c) and Figure S2C(a–c)).

### 3.4. Effects of SFRP1 Stimulation on Odontoblastic Differentiation of hDPCs

**Figure 4:**
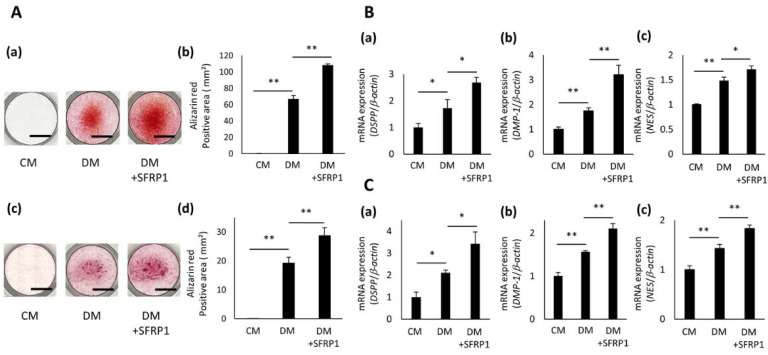
Effects of SFRP1 stimulation on odontoblastic differentiation of hDPCs. (**A**) Odontoblastic differentiation of hDPC-5N and -5I cultured with recombinant SFRP1. Images of Alizarin Red S staining of hDPC-5N (**a**) and -5I (**c**) cultured in CM, DM, or DM with recombinant SFRP1 (100 ng/mL; DM+SFRP1) for 5 days. Experiments were performed in quadruplets. Representative data are shown. The Alizarin Red S-positive area of hDPC-5N (**b**) and -5I (**d**) cultured in CM, DM, or DM+SFRP1 for 5 days. Gene expression of DSPP (**a**), DMP1 (**b**), and NES (**c**) in hDPC-5N (**B**) and -5I (**C**) cultured in CM, DM, or DM+SFRP1 for 3 days. Data are shown as the mean ± standard deviation (*n* = 3). * *p* < 0.05, ** *p* < 0.01. Scale bars = 5 mm.

To determine the effective concentration of SFRP1 for odontoblastic differentiation, hDPC-5I were cultured in CM or DM with various concentrations of SFRP1 for 5 days. SFRP1 promoted the formation of mineralized nodules and increased the Alizarin Red S-positive area in a dose-dependent manner up to 100 ng/mL (Supplemental Figure S3A,B). However, the positive area for Alizarin Red S staining was smaller in cells exposed to DM with 200 ng/mL SFRP1 than that in cells exposed to DM with 100 ng/mL SFRP1. Based on this result, we used 100 ng/mL SFRP1 stimulation in the series of experiments examining odontoblastic differentiation of hDPCs. All three hDPC lines cultured in DM for 5 days generated mineralized nodules, whereas cells cultured in CM formed no nodules (Figure 4A(a,c) and Figure S4A(a)). Moreover, hDPCs exposed to DM+SFRP1 generated more nodules than cells exposed to DM (Figure 4A(a,c) and Figure S4A(a)). The positive area for Alizarin Red S staining was greater in hDPCs exposed to DM+SFRP1 than that in cells exposed to CM or DM (Figure 4A(b,d) and Figure S4A(b)). DM significantly promoted the expression of DSPP, DMP1, and NES in hDPCs, compared with CM (Figure 4B(a–c),C(a–c) and Figure S4B(a–c)). Additionally, DM+SFRP1 induced significant up-regulation of these genes, compared with DM (Figure 4B(a–c),C(a–c) and Figure S4B(a–c)).

### 3.5. Effects of SFRP1 Regulation on BMP-2 Gene Expression in hDPCs

**Figure 5:**
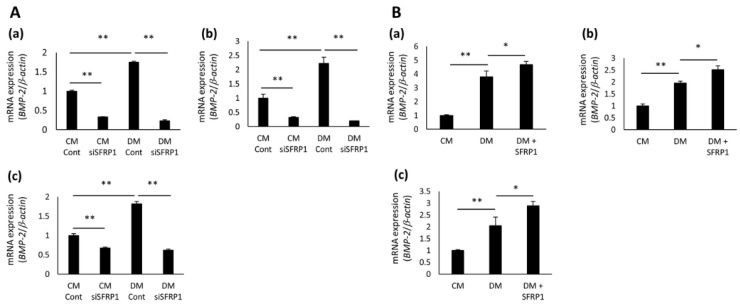
Effects of SFRP1 knockdown and SFRP1 stimulation on BMP-2 gene expression in hDPCs. (**A**,**B**) Gene expression of BMP-2 in hDPCs. Following the transduction of Cont or siSFRP1, hDPCs were cultured in CM or DM for 2 days (**A**). Non-transduced hDPCs were cultured in CM, DM, or DM+SFRP1 for 3 days (**B**). hDPC-5N (**a**), -5I (**b**), and -3R (**c**) were used in this study. Data are shown as the mean ± standard deviation (*n* = 3). * *p* < 0.05, ** *p* < 0.01.

Several studies have suggested that SFRP1 has the potential to regulate BMP signaling [21,22]; therefore, we investigated the gene expression of BMP-2, after SFRP1 knockdown and SFRP1 stimulation in hDPCs. DM significantly enhanced the expression of BMP-2 in Cont- and non-transduced hDPCs compared with CM (Figure 5A(a–c),B(a–c)). However, the expression of BMP-2 was significantly decreased in siSFRP1-transduced hDPCs cultured in CM compared with Cont-transduced cells cultured in CM ((Figure 5A(a–c)). The suppression of BMP-2 expression in siSFRP1-transduced hDPCs was not restored when they were cultured in DM (Figure 5A(a–c)). In contrast, DM+SFRP1 significantly promoted the expression of BMP-2 in non-transduced hDPCs, compared with DM (Figure 5B(a–c)).

### 3.6. Effects of SFRP1 on Reparative Dentin Formation after Direct Pulp Capping Treatment

**Figure 6:**
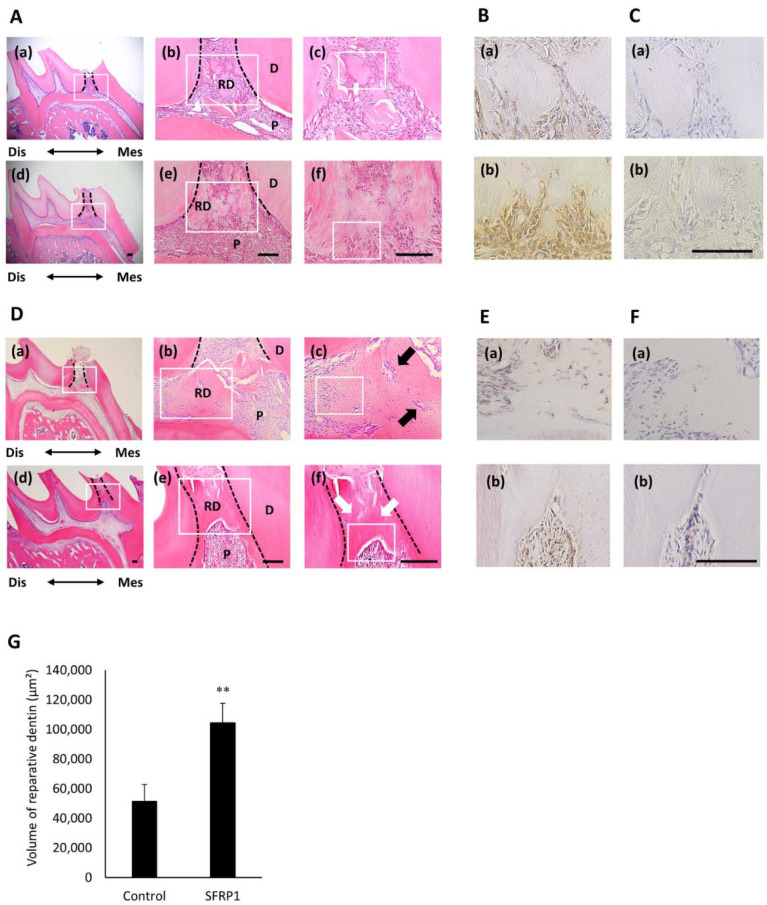
Effects of SFRP1 on reparative dentin formation after direct pulp capping treatment. (**A**) Images of HE-stained maxilla first molars from 10-week-old male Wistar rats after 2 weeks of direct pulp capping treatment. Representative images of specimens applied with nano β-TCP collagen scaffolds containing 0 (**a**–**c**) or 200 ng (**d**–**f**) rhSFRP1. (**b**,**e**) Higher magnification images of white boxes in (**a**,**d**). (**c**,**f**) Higher magnification images of white boxes in (**b**,**e**). Dotted lines indicate a border between primary dentin and newly formed reparative dentin. Immunohistochemical staining for anti-Nes (**B**) and rabbit control IgG (**C**) antibodies in the serial sections of (**A**). These images are higher magnifications of the areas in the white boxes in (**c**,**f**). (**D**) Images of HE-stained maxilla first molars from 12-week-old male Wistar rats after 4 weeks of direct pulp capping treatment. (**b**,**e**) Higher magnification images of white boxes in (**a**,**d**). (**c**,**f**) Higher magnification images of white boxes in (**b**,**e**). Dotted lines indicate a border between primary dentin and newly formed reparative dentin. Black arrows indicate embedded dental pulp cells in generated bone-like dentin. White arrows indicate dentinal tube-like structures in reparative dentin. Immunohistochemical staining for anti-Nes (**E**) and rabbit control IgG (**F**) antibodies in the serial sections of (**D**). These images are higher magnifications of the areas in the white boxes in (**c**,**f**). Representative images of five independent specimens treated with nano β-TCP collagen scaffolds containing 0 (**a**) or 200 ng (**b**) rhSFRP1. (**G**) The amount of newly formed reparative dentin in control and 200 ng rfSFRP1 groups after 4 weeks of direct pulp capping treatment. Data are shown as the mean ± standard deviation (*n* = 5). ** *p* < 0.01. Bars = 100 μm. rhSFRP1, recombinant human SFRP1; Dis, distal; Mes, mesial; D, dentin; P, dental pulp; RD, reparative dentin.

To assess the effects of SFRP1 on reparative dentin formation, we performed direct in vivo pulp capping treatment with SFRP1 recombinant protein. Two weeks after treatment, a small amount of dentin had formed below the pulp exposure site in the control group (Figure 6A(a–c)). In contrast, reparative dentin was generated below the pulp exposure site in the SFRP1 group (Figure 6A(d–f)). The expression of Nes was weak in the control group (Figure 6B(a)); however, its strong expression was confirmed under reparative dentin in the SFRP1 group (Figure 6B(b)). No staining was observed with control IgG (Figure 6C(a,b)). Four weeks after treatment, bone-like dentin was formed below the pulp exposure site and pulp exposure was not closed in the control group (Figure 6D(a–c)). In the SFRP1 group, a greater amount of reparative dentin was generated on the pulp exposure site than in the control group that included dentinal tube-like structures and completely plugged the pulp chamber (Figure 6D(d–f) and Figure S6A). The expression of Nes was faint close to the newly formed reparative dentin in the control group (Figure 6E(a)) but was strong in the SFRP1 group (Figure 6E(b)). No staining was observed with control IgG (Figure 6F(a,b)). The amount of newly formed reparative dentin was quantified using the HE stained samples. It was significantly greater in the SFRP1 group than that of the control group (Figure 6G).

## 4. Discussion

The purpose of direct pulp capping treatment is to maintain pulp health and vitality in an attempt to induce protective reparative dentin formation. While primary dentin is formed by odontoblasts and reparative dentin is produced by odontoblast-like cells [10], these cells have similar characteristics: they are derived from mesenchymal stem cells or progenitor cells in the dental pulp and their primary function is the secretion of dentin during dentinogenesis. Therefore, we hypothesized that signaling molecules that play key roles in odontoblast development have the potential to promote reparative dentin formation by inducing the generation of odontoblast-like cells.

SFRP1 is involved in the development of several tissues; SFRP1 knockout mice show impaired distal lung development and SFRP1 regulates distinct aspects of dopamine neuron development in the midbrain [23,24]. Therefore, we first investigated the expression of Sfrp1 during the development of primary dentin. Our results demonstrated strong expression of Sfrp1 in mature odontoblasts compared with that in cells in the dental pulp, cervical loop, and inner enamel epithelium, and ameloblasts and preodontoblasts. This finding was supported by a previous report that showed strong expression of Sfrp1 in odontoblast-layers of newborn molar tooth germs [17]. These results indicate the crucial roles of SFRP1 in the differentiation of stem/progenitor cells into odontoblasts and in the development of primary dentin.

We also examined the expression of Sfrp1 in mature dental tissues. Stronger Sfrp1 immunostaining was observed in the odontoblast layer than in the pulp, periodontal ligament, cementum, and alveolar bone. Furthermore, SFRP1 expression was higher in hDPCs than in hPDLCs and hGFs. SFRP1 expression was confirmed in cultured rat gingival fibroblasts [25]. Other previous studies demonstrated that bone-lining osteoblast-like stromal cells and osteoclasts express SFRP1 [26] and that SFRP1 is expressed much more strongly in the periodontal ligament than in alveolar bone and cementum [27]. Based on these results, odontoblasts were suggested to express SFRP1 at high levels, not only during primary dentin development but also after its formation was completed. This indicated SFRP1 to be involved in both the development and homeostasis of the dentin-pulp complex.

Various signals, such as growth factors, chemokines, and extracellular matrices, are involved in the regeneration and homeostasis of the dentin-pulp complex [25,26,27,28,29,30]. Additionally, the mesenchymal stem/progenitor cell population in pulp is considered to differentiate into odontoblast-like cells, which generate reparative dentin in response to injury [10,31]. hDPCs were reported to contain stem cell populations that have a high potential to differentiate into odontoblast-like cells [32]. Therefore, we investigated the effects of SFRP1 on the odontoblastic differentiation of hDPCs. We previously demonstrated that CaCl_2_ stimulation induced the differentiation of hDPCs into odontoblast-like cells [20]. Accordingly, CaCl_2_-treated hDPCs formed more mineralized nodules and expressed odontoblast marker genes more highly compared with untreated cells. Intriguingly, SFRP1 expression was also up-regulated in CaCl_2_-treated hDPCs. We also showed that SFRP1 knockdown inhibited CaCl_2_-induced mineralized nodule formation and odontoblast marker gene expression in hDPCs and that SFRP1 treatment enhanced these effects of CaCl_2_ on hDPCs. These results indicated that SFRP1 has the potential to promote the odontoblastic differentiation of stem/progenitor cells in the pulp. However, a previous report demonstrated the opposite result to our findings; some small molecule canonical Wnt signaling agonists promote reparative dentin formation when applied to the surface of artificially exposed pulp in vivo [33]. SFRP1 is a well-known canonical Wnt signaling inhibitor because it binds to Wnt ligands preventing their binding to Frizzled receptors and/or form signaling-inactive complexes with these receptors [15]. Interestingly, SFRP1 knockdown did not induce the translocation of β-catenin into nuclei (Supplemental Figure S5A). Moreover, SFRP1 stimulation did not alter the expression of active β-catenin in hDPC-5I (Supplemental Figure S5B). These results suggested that SFRP1 was not involved in the alteration of Wnt signaling in hDPCs unlike Wnt signaling agonists. Sugiyama et al. also suggested that SFRP1 does not act as the putative negative regulator that blocks Wnt signaling during lens induction [34]. Rodrigues et al. demonstrated that SFRP1 can regulate the growth of retinal ganglion cell growth cones without Wnt signaling inhibition [35] and revealed that repression of androgen receptors by SFRP1 did not require sequestration of Wnt signals [36]. These reports strongly support our result of SFRP1. Taken together, the findings by ourselves and others indicate that SFRP1 regulates odontoblastic differentiation of hDPCs by controlling signaling pathways other than canonical Wnt signaling.

SFRP1 interacts with various signaling pathways, including Hedgehog, TGF, Notch, and BMP [22,37]. BMP-2 is a member of TGF-βsuperfamily and has crucial roles in the development, remodeling, and homeostasis of bone and cartilage [38]. BMP-2 expression is also identified in dental cells during tooth development [39] and promotes odontoblastic differentiation of dental pulp stem cells via their commitment to odontoblast lineages [40,41]. Moreover, BMP-2 conditional knock-out mice exhibited delayed odontoblast differentiation, abnormal dentin tubule formation, and low odontoblast-related gene expression [42,43] and BMP2-transfected dental pulp stem cells promoted hard tissue formation and odontoblast-related gene expression [40], suggesting its important functions in dentinogenesis. These reports are consistent with our results of increased BMP2 expression in CaCl_2_-treated hDPCs, compared with that in untreated cells. We also revealed that SFRP1 knockdown inhibited, while SFRP1 treatment enhanced, this CaCl_2_-induced increase in expression. These results indicate that SFRP1 is involved in dentinogenesis by regulating BMP-2 expression in dental pulp stem/progenitor cells to induce their odontoblast-like cell differentiation.

Based on the ability of SFRP1 to promote odontoblastic differentiation of hDPCs in vitro, we next aimed to evaluate its effects on the formation of reparative dentin and the preservation of pulp integrity using the in vivo direct pulp capping treatment model. Scaffolds with 200 ng rhSFRP1 considerably induced the formation of mineralized tissues at the site of pulp exposure. Interestingly, they completely plugged pulp exposure. The formation of reparative dentin was also observed with the indication of 100 ng of rhSFRP1, however closure of the exposed pulp was not confirmed (Supplemental Figure S6C(a–c)). These results suggest that rhSFRP1 induces reparative dentin formation in vivo in a dose-dependent manner. Interestingly, dentin-like hard tissues were formed below the pulp exposure site and the pulp exposure was closed after direct pulp capping with MTA (Supplemental Figure S6D(a–c)). However, these hard tissues should be different from the reparative dentin that rhSFRP1 induced to form because they did not have a tube-like structure and included many voids. The result was consistent with the previous report that hard tissues derived from MTA did not resemble reparative dentin because they contain porosities and tunnel defects [9]. On the other hand, the reparative dentin formed in SFRP1 group had almost no voids or embedded cells unlike control group and MTA capping group, and had tubule-like structures similar to dentinal tubules in primary dentin (Figure 6D(d–f) and Figure S6A).

The expression of Nes was low in the dentin-pulp complexes of the control group but was strong beneath the reparative dentin in the SFRP1 group after 2 and 4 weeks of direct pulp capping treatment. Nes is exclusively expressed in functioning odontoblasts of adult teeth; however, its expression disappeared in injured odontoblasts and subsequently emerged in newly generated odontoblast-like cells [44]. Furthermore, the expression of Dsp was confirmed in order to characterize reparative dentin. Dsp expression on reparative dentin generated in SFRP1 group was stronger than control group (Supplemental Figure S6B(a,b)). This result indicates a greater generation of odontoblast-like cells during the repair of dental pulp injury in the SFRP1 group compared with that in the control group.

A previous report demonstrated that SFRP1 knockout mice showed the up-regulation of bone mass compared with wild type mice [45]. This finding was opposite to our result of SFRP1 revealing the promotive effect on reparative dentin formation. Odontoblasts resemble osteoblasts because they have the potential to form mineralized tissue. However, some factors like DSPP and DMP-1 were known to be expressed in odontoblasts compared with osteoblasts, suggesting that they have different phenotypes. Moreover, several molecules were reported to show dualistic effects: basic fibroblast growth factor promoted mineralized nodule formation of mature osteoblasts, yet suppressed osteoblastic differentiation of immature ones [46]. While Periostin played important roles in the differentiation of osteoblasts, it acted as a negative regulator in the odontoblastic differentiation of dental pulp cells [47]. These results indicated that SFRP1 shows a dualistic effect depending on the cell types. Further research is essential to clarify that the function of SFRP1 differs between osteoblasts and odontoblasts.

Nano β-TCP collagen scaffolds were used in this study because our previous report demonstrated that they could retain Semaphorin 3A protein and promoted reparative dentin formation in in vivo direct pulp capping treatment model [13]. However, various types of scaffolds and stem cell interactions also have been reported: human periapical cyst mesenchymal stem cells cultured on polylactic acid scaffolds containing dicalcium phosphate dihydrate and hydraulic calcium silicate showed high proliferative ability and promoted DMP-1 gene expression [48]. Human dental pulp stem cells seeding on silicon discs with various sizes of porous increased proliferation, compared with cells on silicon discs without porous [11]. In addition, acellular scaffolds consisting of organic components of the extracellular matrix have shown great potential in producing functional tissues. Aulino et al. reported that implanted skeletal muscle acellular scaffolds promoted colonization of stem cells in muscle and bone tissues [49]. These reports suggest that providing a conducive environment for stem cells should be important to realize tissue regeneration. Therefore, further studies are necessary to identify a more appropriate scaffolding material for sFRP1 in the direct pulp capping treatment.

In periodontal treatment, proteins such as enamel matrix proteins [50] and bFGF [51] have been clinically applied for the purpose of regenerating periodontal tissue destroyed by severe periodontitis. Therefore, it is considered that applying signal factor protein to dental pulp treatment is very effective. In endodontic treatment research, direct pulp capping has been attempted by applying growth factors present in bone and dentin matrices such as BMPs and TGFβ [52,53], but a porous bone-like structure was generated just below the pulp capping surface and reparative dentin similar to primary dentin was not induced. Our result indicated that SFRP1 induced reparative dentin with a tubule-like structure similar to primary dentin. It is expected that SFRP1 can be a useful material for preserving dental pulp tissue by direct pulp capping.

## 5. Conclusions

SFRP1 is strongly expressed in odontoblasts during and after odontogenesis. SFRP1 knockdown suppresses odontoblastic differentiation of hDPCs and SFRP1 stimulation promotes their differentiation via the regulation of BMP-2 expression. In direct pulp capping models, SFRP1 induces reparative dentin formation at the site of pulp exposure. The application of SFRP1 would be a breakthrough treatment for exposed dental pulp because it has the potential to induce reparative dentin formation, which is based on dentinogenesis. Direct pulp capping material containing SFRP1 may be a promising alternative to conventional pulp capping materials.

## Figures and Tables

**Table 1:** Specific primer sequences, annealing temperature, cycle numbers, product sizes, and sequence IDs for semi-quantitative RT-PCR.

Target Gene (Abbreviation)	Forward (Top) and Reverse (Bottom) Primer Sequences	Size of Amplified Products (bp)	Annealing Temperature (°C)	Cycles	Sequence ID
*SFRP1*	AAAGCAAGGGCCATTTAGATTAG TTCTGGGCTTGACCTTAATTGTA	328	55	27	NM_003012.5
*GAPDH*	ACCACAGTCCATGCCATCCAC TCCACCACCCTGTTGCTGTA	452	60	18	NM_001256799.2

**Table 2:** Specific primer sequences, annealing temperature, cycle numbers, product sizes, and sequence IDs for quantitative RT-PCR.

Target Gene (Abbreviation)	Forward (Top) and Reverse (Bottom) Primer Sequences	Size of Amplified Products (bp)	Annealing Temperature (°C)	Cycles	Sequence ID
*SFRP1*	CAAGAAGAAGAAGCCCCTGA AAGTGGTGGCTGAGGTTGTC	123	60	40	NM_003012.5
*DSPP*	ATATTGAGGGCTGGAATGGGGA TTTGTGGCTCCAGCATTGTCA	136	60	40	NM_014208.3
*DMP-1*	CCCTTGGAGAGCAGTGAGTC CTCCTTTTCCTGTGCTCCTG	166	60	40	NM_004407.4
*NES*	TGGCCACGTACAGGACCCTCC AGATCCAAGACGCCGGCCCT	143	60	40	NM_006617.1
*BMP-2*	TCCACTAATCATGCCATTGTTCAGA GGGACACAGCATGCCTTAGGA	74	60	40	NM_001200.4
β*-actin*	ATTGCCGACAGGATGCAGA GAGTACTTGCGCTCAGGAGGA	89	60	40	NM_001101.3

## Supplementary Materials

The following are available online at https://www.mdpi.com/article/10.3390/cells10092491/s1, Supplemental Figure S1 Gene expression of *SFRP1* during odontoblastic differentiation of hDPC-3R, Supplemental Figure S2 Effects of *SFRP1* down-regulation on odontoblastic differentiation in hDPC-3R, Supplemental Figure S3 Effects of various concentration of SFRP1 stimulation on odontoblastic differentiation of hDPC-5I, Supplemental Figure S4 Effects of SFRP1 stimulation on odontoblastic differentiation of hDPC-3R, Supplemental Figure S5 Effects of *SFRP1* knockdown and SFRP1 stimulation on β-catenin activation and nuclear transduction in hDPC-5I, Supplemental Figure S6 Direct pulp capping treatment with SFRP1 and MTA.
