# Supplementary material for: Secreted Frizzled-Related Protein 1 Promotes Odontoblastic Differentiation and Reparative Dentin Formation in Dental Pulp Cells
Source: Cells. 2021 Sep 21;10(9):2491. doi: 10.3390/cells10092491 (PMC8468928; doi:10.3390/cells10092491)
Supplement: Supplementary file 1 [file cells-10-02491-s001.zip › cells-1357650-Supplemental figures.pdf]

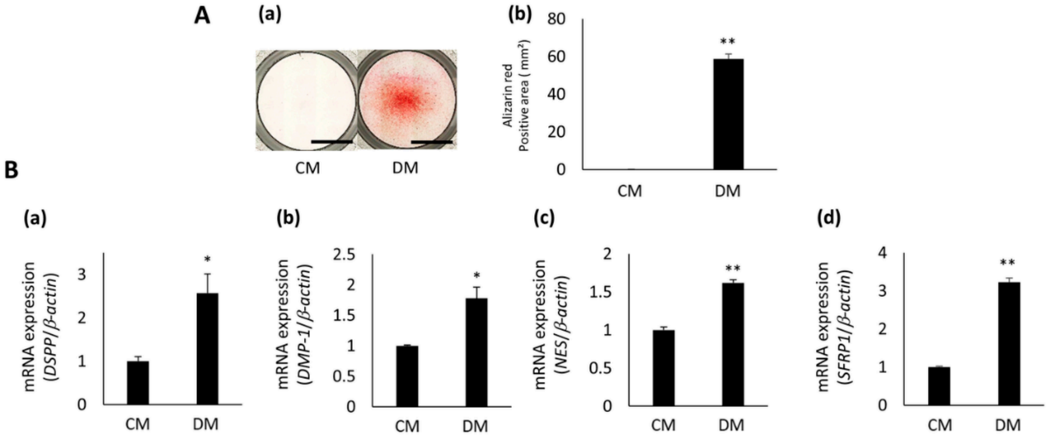

Supplemental figure. S1 Gene expression of *SFRP1* during odontoblastic differentiation of hDPC-3R

(A) Odontoblastic differentiation of hDPC-3R. (a) Alizarin Red S staining images of hDPC-3R cultured in control culture medium (CM) or odontoblastic differentiation medium (DM) for 5 days. Experiments were performed in quadruplet. Representative data are shown. (b) The Alizarin Red S-positive area of hDPC-3R cultured in CM or DM for 5 days. (B) Gene expression of odontoblast related-markers, *DSPP* (a), *DMP1* (b) , *NES* (c) and *SFRP1* (d) in hDPC-3R cultured in CM or DM for 3 days. Data are shown as the mean ± standard deviation (n=3). *p < 0.05, **p < 0.01. Scale bars = 5 mm.

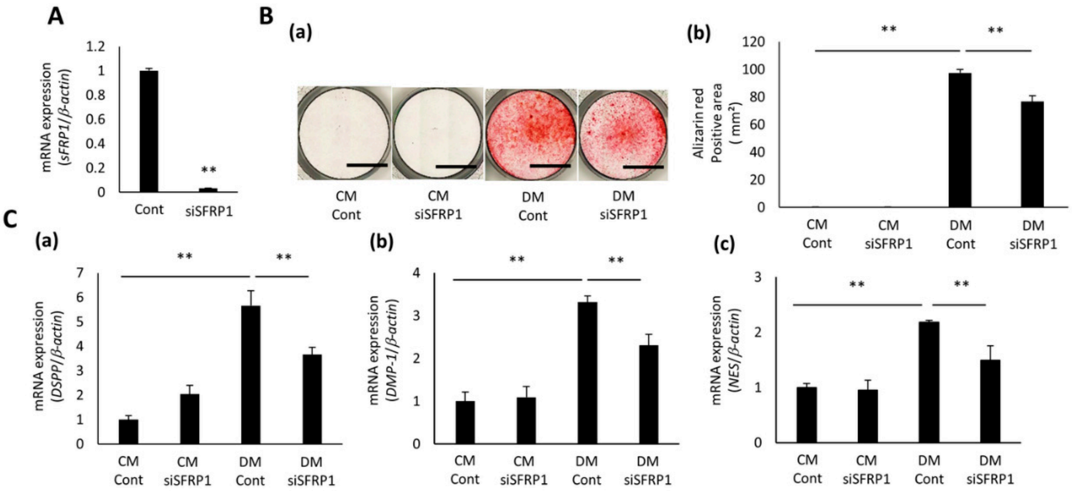

Supplemental figure. S2 Effects of *SFRP1* down-regulation on odontoblastic differentiation in hDPC-3R

(A) *SFRP1* gene expression in hDPC-3R transduced with control siRNA (Cont) or SFRP1 siRNA (siSFRP1). (B) Odontoblastic differentiation of siRNA-transduced hDPC-3R. (a) Images of Alizarin Red S staining of siRNA-transduced hDPC-3R cultured in CM or DM for 5 days. Experiments were performed in quadruplet. Representative data are shown. (b) The Alizarin Red S-positive area of siRNA-transduced hDPC-3R cultured in CM or DM for 5 days. (C) Gene expression of *DSPP* (a), *DMP1* (b) and *NES* (c) in siSFRP1-transduced

hDPC-3R cultured in CM or DM for 2 days. Data are shown as the mean ± standard deviation (n=3). *p < 0.05, **p < 0.01. Scale bars = 5 mm.

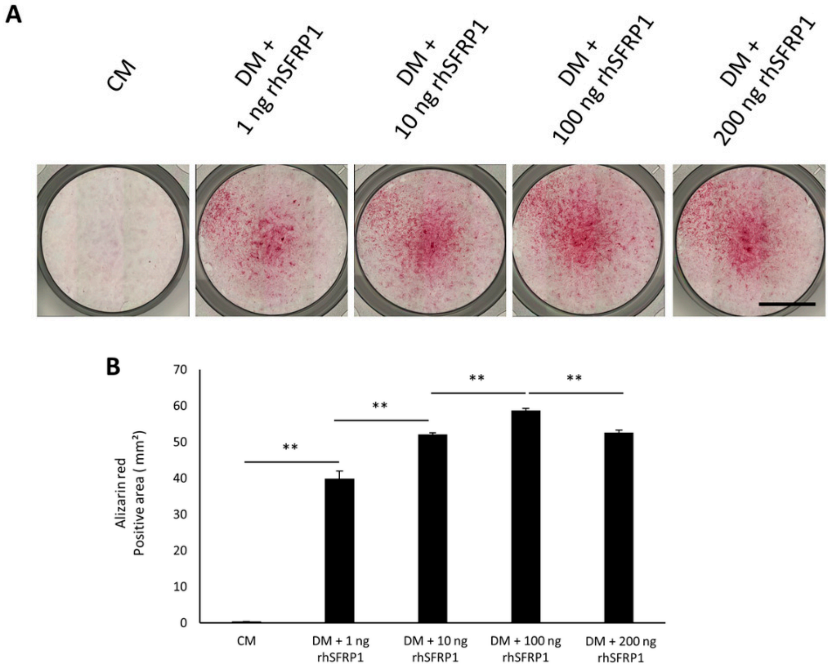

Supplemental figure. S3 Effects of various concentration of SFRP1 stimulation on odontoblastic differentiation of hDPC-5I

(A) Images of Alizarin Red S staining of hDPC-5I cultured in CM or DM with recombinant SFRP1 (1, 10, 100 or 200 ng/ml) for 5 days. Experiments were performed in quadruplet. Representative data are shown. (B) The Alizarin Red S-positive area of hDPC-5I cultured in CM or DM with recombinant SFRP1 for 5 days. **p < 0.01. Scale bars = 5 mm.

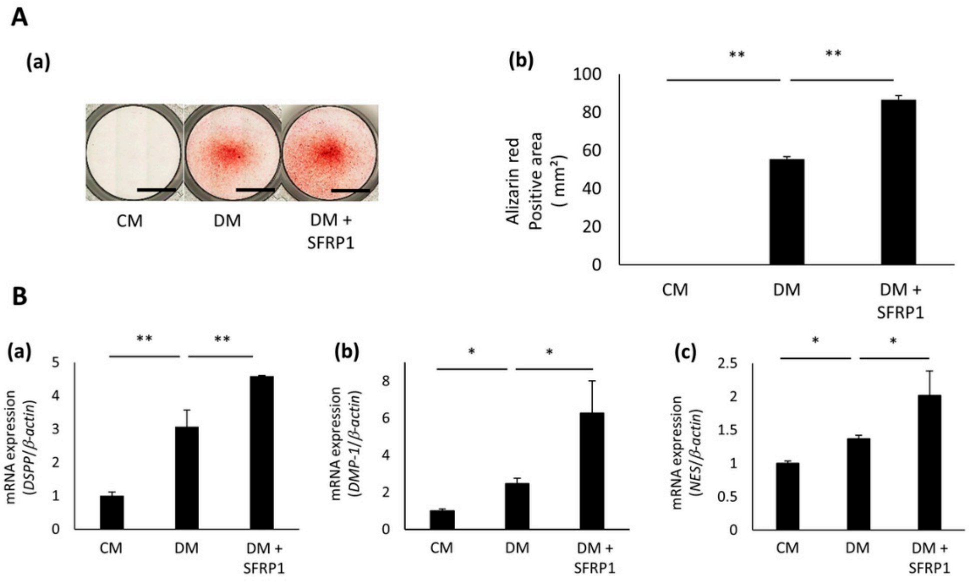

Supplemental figure. S4 Effects of SFRP1 stimulation on odontoblastic differentiation of hDPC-3R

(A) Odontoblastic differentiation of hDPC-3R cultured with recombinant SFRP1. (a) Images of Alizarin Red S staining of hDPC-3R cultured in CM, DM or DM+SFRP1 for 5 days. Experiments were performed in quadruplet. Representative data are shown. (b) The Alizarin Red S-positive area of hDPC-3R cultured in CM, DM or DM+SFRP1 for 5 days. (B) Gene expression of *DSPP* (a), *DMP1* (b) and *NES* (c) in hDPC-3R cultured in CM, DM or DM+SFRP1 for 3 days. Data are shown as the mean ± standard deviation (n=3). *p < 0.05, **p < 0.01. Scale bars = 5 mm.

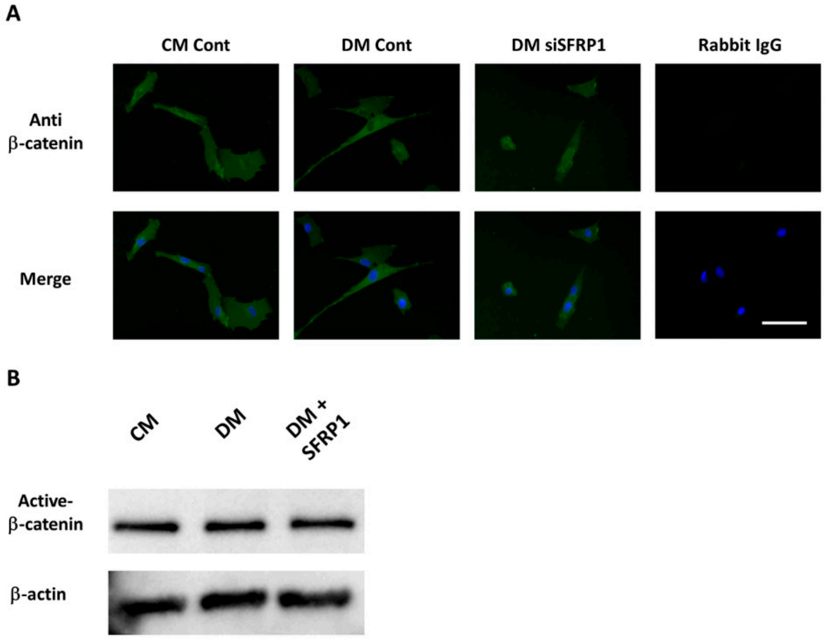

Supplemental figure. S5 Effects of *SFRP1* knockdown and SFRP1 stimulation on β-catenin nuclear transduction and activation in hDPC-5I

(A) Immunofluorescence staining for anti-β-catenin and rabbit control IgG antibodies in siRNA-transduced hDPC-5I cultured in CM or DM for 1 day. Nuclei were stained with DAPI. Scale bars = 50 mm. (B) Changes in active-β-catenin and β-actin protein levels in hDPC-5I that were cultured in CM, DM or DM+SFRP1 for 3 days.

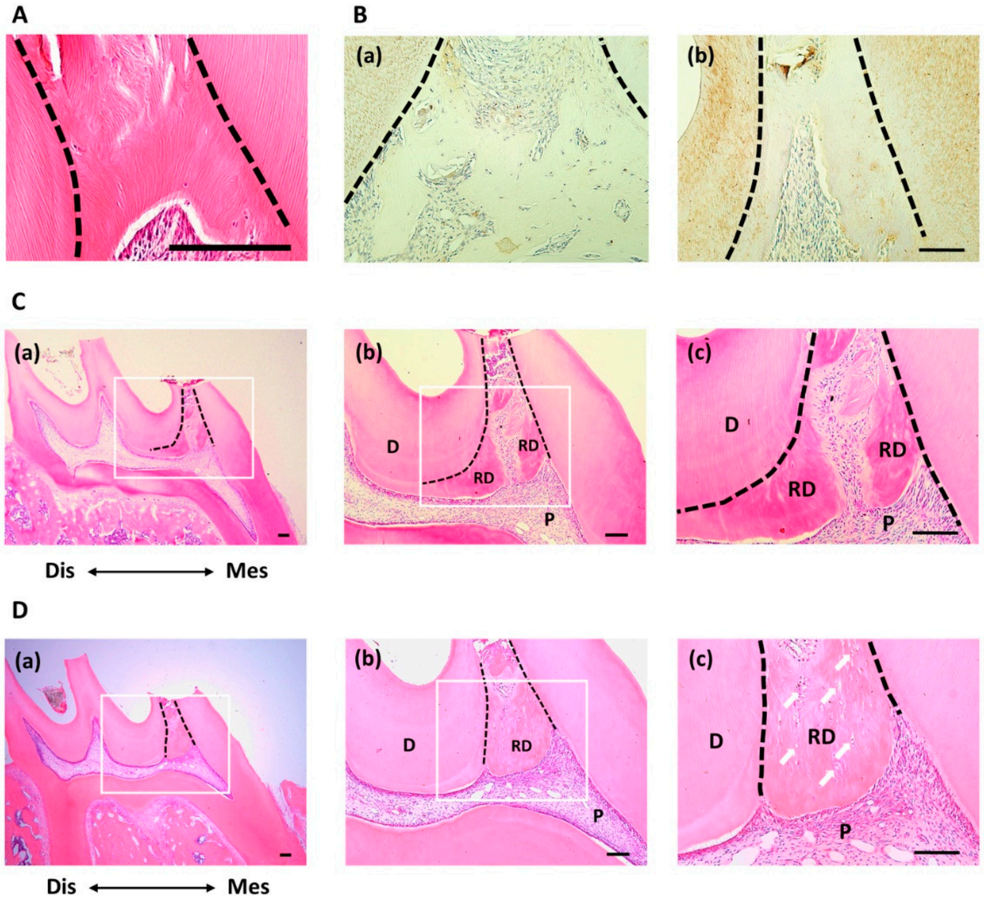

Supplemental figure. S6 Direct pulp capping treatment with SFRP1 and MTA

(A) Higher magnification images of reparative dentin induced with 200 ng rhSFRP1. (B) Immunohistochemical staining for anti-Dsp antibody in the serial sections of applied with nano β-TCP collagen scaffolds containing 0 (a) or 200 ng (b) rhSFRP1. Images of HE-stained maxilla first molars from 12-week-old male Wistar rats after 4 weeks of direct pulp capping treatment with 100 ng rhSFRP1 (C) and MTA (D). (b) Higher magnification images of white boxes in (a). (c) Higher magnification images of white boxes in (b). Dotted lines indicate a border between primary dentin and newly-formed reparative dentin. Arrows indicate porosities in reparative dentin. Bars = 100 μm. rhSFRP1, recombinant human SFRP1; Dis, distal; Mes, mesial; D, dentin; P, dental pulp; RD, reparative dentin.
